# Zoonotic Leishmaniasis, Bosnia and Herzegovina

**DOI:** 10.3201/eid2502.181481

**Published:** 2019-02

**Authors:** Vito Colella, Adnan Hodžić, Roberta Iatta, Gad Baneth, Amer Alić, Domenico Otranto

**Affiliations:** Università degli Studi di Bari, Bari, Italy (V. Colella, R. Iatta, D. Otranto);; University of Veterinary Medicine Vienna, Vienna, Austria (A. Hodžić);; The Hebrew University of Jerusalem, Rehovot, Israel (G. Baneth);; University of Sarajevo, Sarajevo, Bosnia and Herzegovina (A. Alić)

**Keywords:** Leishmania infantum, leishmaniasis, zoonoses, parasites, neglected diseases, dogs, cats, Bosnia and Herzegovina, eastern Europe

## Abstract

*Leishmania infantum* causes potentially life-threatening disease in humans. To determine the extent of the animal reservoir for this pathogen in Bosnia and Herzegovina, we tested dogs and cats. We found that a large proportion of dogs were exposed to or infected with *L. infantum*, indicating endemicity in dogs and zoonotic risk for humans.

Among neglected tropical diseases, visceral leishmaniasis is one of the most deadly parasitic diseases in modern history. Worldwide, it causes an estimated 300,000 new cases and ≈20,000 deaths each year (*1*). Leishmaniasis has been the hallmark of poverty-related diseases and of tropical infections in humans forced to migrate from and to conflict areas (*1*).

Like other countries in the Balkan area, Bosnia and Herzegovina is considered a hotspot for neglected infections of poverty (*2*). The economic, political, and social transformations of this country reflect the armed conflicts of the recent past; 16.9% of the population lives under the national poverty level (*3*). In Bosnia and Herzegovina, leishmaniasis is considered hypoendemic, possibly because of lack of awareness among medical personnel (*4*). Although the first autochthonous cases of leishmaniasis in Bosnia and Herzegovina (in 4 children) occurred in 1949 and 1954 (*5*), during the past 10 years, only 7 patients in the capital city of Sarajevo have been hospitalized for this disease (*4*).

In 2013, 8.2% of the soldiers of the Austrian Armed Forces deployed in peacekeeping missions in Bosnia and Herzegovina were exposed to *Leishmania* spp. (*6*). Despite small increases in reports of *Leishmania infantum* infection in human patients from Bosnia and Herzegovina, little information is available about dogs as reservoirs, the main recognized reservoir of zoonotic infection and the target of integrated control strategies. To determine the role of animals in the spread of leishmaniasis, we assessed their exposure to and infection with *L. infantum*. The study protocol was approved by the Ethical Committee of the Department of Veterinary Medicine of the University of Bari (Prot. UniBa 11/18).

In 2017, we tested blood samples from 408 dogs and 5 cats from different areas of Bosnia and Herzegovina for leishmaniasis (Table). To assess the exposure of dogs to *Leishmania* spp., we tested 180 dogs (free-roaming, client-owned, and shelter) for *L. infantum* antibodies by using an immunofluorescence antibody test (*7*); we tested all 408 dogs and 5 cats for the parasites’ DNA by using real-time PCR (*8*). Samples that were positive by real-time PCR were further analyzed by conventional PCR for sequencing confirmation of the kinetoplast DNA (*9*) and of the partial internal transcribed spacer 2 ribosomal RNA (*10*). 

**Table:** Positivity to *Leishmania infantum* of dogs and cats kept in different living conditions and from selected geographic areas in Bosnia and Herzegovina*

Animal characteristic	IFAT positive/total (%)	Real-time PCR positive/total (%)
All tested	30/180 (16.7)	13/413 (3.1)
Living condition		
Shelter	14/96 (14.6)	0/142
Stray	10/65 (15.3)	3/127 (2.4)
Client-owned	6/19 (31.6)	10/134 (7.5)
Geographic origin		
Sarajevo	1/22 (4.5)	7/142 (4.9)
Gračanica	2/56 (3.5)	0/63
Zenica	9/39 (23.1)	0/44
Goradže	12/34 (35.3)	0/40
Bihać	0	0/40
Mostar	4/6 (66.7)	6/36 (16.7)
Livno	2/14 (14.3)	0/14
Odžak	ND	0/12
Gornji Vakuf-Uskoplje	ND	0/12
Tuzla	0/9	0/9

*IFAT, immunofluorescence antibody test; ND, not done.

Overall, we obtained positive results for 16.7% (95% CI 11.2%–22%) of dogs by immunofluorescence antibody test and 3.1% (95% CI 1.5%–4.8%) of dogs by PCR (Table), indicating that a large proportion of the canine population from Bosnia and Herzegovina has been exposed to or is infected by *L. infantum*. In addition, we obtained positive results for a client-owned cat from Sarajevo tested by real-time PCR. Consensus sequences of the partial internal transcribed spacer 2 ribosomal RNA and kinetoplast DNA from all animals with positive test results displayed 100% identity to the nucleotide sequences of *L. infantum* and are available in GenBank (accession nos. MH605316 and MH605317).

After World War II, leishmaniasis was frequently reported for humans and dogs in countries bordering Bosnia and Herzegovina, such as Croatia and Serbia (*4*), whereas no information was available for leishmaniasis in dogs in Bosnia and Herzegovina. Our findings indicate the presence of *L. infantum* in the canine reservoir population in Bosnia and Herzegovina and support the pathogen’s spread across the country and the consequent infection of soldiers deployed in this region and persons living in this resource-limited setting. Although infections in children and soldiers from Sarajevo and its neighboring municipalities in central Bosnia (i.e., Fojnica, Kakanj, and Gornji Vakuf-Uskoplje) have been reported, we show that *L. infantum*–infected dogs are broadly distributed throughout the country, indicating a potential risk for more human infections. 

In Bosnia and Herzegovina, diagnosis and treatment of leishmaniasis is possible only in specialized hospitals, which entails admission of patients at a progressive and often lethal stage of the disease (*4*). The World Health Organization prioritizes efforts to control or eliminate leishmaniasis in countries where the incidence of the infection in humans is high and advocates for passive surveillance in countries where leishmaniasis is not endemic. Although these efforts are leading to large improvements in the control of the disease in humans, this direction is not optimal for unraveling and fighting neglected leishmaniasis in low-resource settings where no information about animal reservoirs is available, such as Bosnia and Herzegovina. The high percentage and wide distribution of dogs exposed to *L. infantum* infections implies the existence of autochthonous transmission in the country, thus suggesting that so far, zoonotic leishmaniasis has been neglected in Bosnia and Herzegovina.

Although Bosnia and Herzegovina is located near wealthy countries (Europe includes most of the highly industrialized G8 nations), it ranks in the last quartile of the global human developmental index. Hence, a greater political commitment of European Union policy makers is central to implementation of a proper surveillance system to promptly identify animal reservoirs of *L. infantum* and favor timely diagnosis and treatment for human populations living in poverty and for domestic animals. These data should encourage a One Health approach to provide comprehensive tools and policy recommendations to fight neglected leishmaniasis in Bosnia and Herzegovina.
